# Influence of data quality on computed Dutch hospital quality indicators: a case study in colorectal cancer surgery

**DOI:** 10.1186/1472-6947-14-32

**Published:** 2014-04-11

**Authors:** Kathrin Dentler, Ronald Cornet, Annette ten Teije, Pieter Tanis, Jean Klinkenbijl, Kristien Tytgat, Nicolette de Keizer

**Affiliations:** 1 Department of Computer Science, VU University Amsterdam, Amsterdam, Netherlands; 2 Department of Medical Informatics, Academic Medical Centre, University of Amsterdam, Amsterdam, The Netherlands; 3 Gastrointestinal Oncology Centre Amsterdam, Academic Medical Centre, University of Amsterdam, Amsterdam, The Netherlands; 4 Department of Biomedical Engineering, Linköping University, Linköping, Sweden

**Keywords:** Data quality, Clinical quality indicators, Electronic medical record, Clinical audit, Patient data, Reuse, Secondary use

## Abstract

**Background:**

Our study aims to assess the influence of data quality on computed Dutch hospital quality indicators, and whether colorectal cancer surgery indicators can be computed reliably based on routinely recorded data from an electronic medical record (EMR).

**Methods:**

Cross-sectional study in a department of gastrointestinal oncology in a university hospital, in which a set of 10 indicators is computed (1) based on data abstracted manually for the national quality register Dutch Surgical Colorectal Audit (DSCA) as reference standard and (2) based on routinely collected data from an EMR. All 75 patients for whom data has been submitted to the DSCA for the reporting year 2011 and all 79 patients who underwent a resection of a primary colorectal carcinoma in 2011 according to structured data in the EMR were included. Comparison of results, investigating the causes for any differences based on data quality analysis. Main outcome measures are the computability of quality indicators, absolute percentages of indicator results, data quality in terms of availability in a structured format, completeness and correctness.

**Results:**

All indicators were fully computable based on the DSCA dataset, but only three based on EMR data, two of which were percentages. For both percentages, the difference in proportions computed based on the two datasets was significant.

All required data items were available in a structured format in the DSCA dataset. Their average completeness was 86%, while the average completeness of these items in the EMR was 50%. Their average correctness was 87%.

**Conclusions:**

Our study showed that data quality can significantly influence indicator results, and that our EMR data was not suitable to reliably compute quality indicators. EMRs should be designed in a way so that the data required for audits can be entered directly in a structured and coded format.

## Background

Over the last decades, it became possible and increasingly interesting to measure the quality of health care to implement quality improvement activities and to strengthen both transparency and accountability [1]. In this context, both legally mandatory and voluntary quality indicators [2] for various kinds of diseases and interventions have been released by governments, patient and scientific associations as well as insurance companies. The computed results are used for performance comparisons between health care institutions. As such comparisons have potentially serious implications, including influencing the choices of patients and insurance companies, indicator results should be reliable.

Ideally, clinical quality indicators are computed inside hospitals based on data recorded during the care process and stored in the Electronic Medical Record (EMR). In the United States, the meaningful use [3] of EMRs is put forward as a national goal, which includes the electronic exchange of health information as well as the computation and reporting of clinical quality measures [4]. This meaningful use reduces the registration burden for care providers and furthermore enables the unobtrusive measuring and monitoring of indicators in real-time, allowing for timely intervention.

Next to this development, national and international medical data registries proliferate [5], which are frequently used to quantitatively compare performance between health-care institutions. Due to various barriers that impede the reuse of data [6], many care organisations still collect the data for quality registers manually [7]. This labour-intensive process might lead to the undesirable situation that the data in registers differs from source data in an EMR.

In the Netherlands, “Zichtbare Zorg” [8] developed amongst others a set of 11 evidence-based colorectal cancer surgery indicators, which is computed based on the register of the Dutch Surgical Colorectal Audit (DSCA) [9]. The DSCA has been set up in 2009 to measure and to improve the quality of colorectal cancer surgery, serving as both national and international role model. All Dutch hospitals that perform colorectal cancer surgery submit data to the DSCA register. Ideally, data should be submitted (semi-)automatically, but in practice surgeons often enter it manually via a web form. The data is often submitted at the end of a reporting year, impeding timely feedback.

This study aims to assess whether the set of quality indicators can be computed automatically based on EMR data and to investigate barriers to succeed. Hence, we compared quality indicators computed based on our EMR data to the same indicators computed based on manually abstracted data for the DSCA register, and performed a data quality analysis to explain any differences.

## Methods

### Patient data

We used two data sources of a department of colorectal cancer surgery in a university hospital: manually abstracted data for the DSCA register and structured data from the EMR.

The DSCA dataset consists of 212 variables, including demographic information, diagnoses, procedures, results of pathological examinations and clinical outcome. Attending surgeons enter the required data, either manually with the help of a web form, which takes 15 to 20 minutes per patient, or with a spreadsheet. In most hospitals the data is entered via the web form. In our hospital, the responsible surgeon preselects the patients for whom to submit data from the database containing all surgical procedures. He then browses structured and unstructured data such as pathology reports for the respective patients to identify as many of the required variables as possible. All patients of our hospital for whom data has been submitted to the DSCA in 2011 were included.

For this study, we regarded the DSCA dataset as the current reference standard. We deliberately do not refer to it as gold standard because we cannot exclude all possibility of errors due to manual data entry. However, surgeons have reported to enter the data carefully. Also, the data is monitored by the DSCA by an annual comparison to the dataset of the Dutch Cancer Registry. Its reliability seems to be high: A recent comparison showed that data has been submitted to the DSCA register for 94% of the patients in the Dutch Cancer Registry. Most data items correspond well, with discrepancies being mainly due to differing interpretations and definitions [10]. For example, anastomotic leakages are only registered in the DSCA if they caused a re-intervention, while the Dutch Cancer Registry handles a broader definition.

Regarding our EMR, several source systems that contain information on patients, diagnoses, operations, admissions, encounters, pathology reports, endoscopies and medications periodically insert data into our data warehouse. Diagnoses are encoded in ICD-9-CM, and surgical procedures in codes from a Dutch procedure classification consisting of nearly 40,000 codes. All patients who had an operation in 2011 have been extracted from the data warehouse. In the following, we refer to this dataset as EMR. All patients from the EMR who seemingly should have been submitted to the DSCA in the reporting year 2011 due to a recorded surgical resection of a primary colorectal carcinoma were included.

#### Patient matching

In absence of patient identifiers, the patients for whom data has been submitted by our hospital to the DSCA in 2011 are matched with the patients from the EMR based on their gender, year of birth and operation date as well as sets of procedures that they underwent.

The Institutional review board of the Academic Medical Centre at the University of Amsterdam waived the need for informed consent, as individual patients were not directly involved. The use of the data is officially registered according to the Dutch Personal Data Protection Act.

### Quality indicators and their computation

We used the set of colorectal quality indicators released by a governmental quality of care program called “Zichtbare Zorg” for the reporting year 2011. The set consists of 8 thematic indicators, 3 of which comprise two related indicators denoted as e.g. 8a and 8b, resulting in a total of 11 indicators: 9 process indicators, 1 structure indicator and 1 outcome indicator (see Table 1). The process and outcome indicators are percentages computed based on the definitions for numerators and denominators of each indicator. The structure indicator 8a (“How many surgeons does the team include and how many of these surgeons carry out resections on primary colonic carcinoma patients?”) is not designed to be computable based on the EMR. Therefore, we did not include it in our study. Of the remaining 10 indicators, the DSCA indicator 1 and the circumferential resection margin indicator 6a measure the percentage of patients for whom data has been submitted to the DSCA. As we do not expect submission of data to the DSCA to be recorded in the EMR, we exclude the numerators of these indicators. The 8 fully and 2 partially (i.e. only the denominator) included indicators have been formalised with our previously developed indicator formalisation method CLIF [11] to enable their automated computation, for which the obtained queries are run against the respective datasets. The queries are published on figshare [12].

**Table 1:** Zichtbare Zorg indicators for 2011 translated from Dutch to English

**1.**	**Dutch Surgical Colorectal Audit (Process)**
*Numerator*	Number of surgical resections of colorectal carcinomas located in colon or rectum (only count resections for primary carcinomas)
	for which data has been submitted to the Dutch Surgical Colorectal Audit
*Denominator*	Number of surgical resections of colorectal carcinomas located in colon or rectum (only count resections for primary carcinomas)
*Inclusion*	Primary carcinomas
*Exclusion*	Recurrent colorectal carcinomas; TEM-resection (transanal endoscopic microsurgery)
**2.**	**Number of lymph nodes examined after resection (Process)**
*Numerator*	Number of patients who had 10 or more lymph nodes examined after resection of a primary colonic carcinoma
*Denominator*	Number of patients who underwent resection of a primary colonic carcinoma
*Inclusion*	All primary carcinomas, for which a part of the colon has been resected via open or laparoscopic surgery
*Exclusion*	1) patients who had a ‘resection’ via colonoscopy; 2) patients with previous radiotherapy; 3) patients with a recurrent carcinoma
**3.**	**Patients with rectum carcinoma discussed in multidisciplinary meeting before surgery (Process)**
*Numerator*	Number of patients with rectum carcinoma who have been discussed in a multidisciplinary meeting before the surgery
*Denominator*	Number of patients with rectum carcinoma operated in reporting year
*Inclusion*	All patients who underwent a resection of rectum due to a primary rectum carcinoma in the reporting year, via open or
	laparoscopic surgery
*Exclusion*	TEM-resections and recurrent rectum carcinoma
**4.**	**Preoperative imaging colon (Process)**
*Numerator*	Number of patients with diagnosed colorectal carcinoma which has been resected electively en whose colon has been imaged
	completely before the surgery
*Denominator*	Number of patients with diagnosed colorectal carcinoma which has been resected electively
*Inclusion*	All primary carcinomas, for which a part of the colon has been resected via open or laparoscopic surgery
*Exclusion*	1) patients who had a ‘resection’ via colonoscopy; 2) patients with previous radiotherapy; 3) patients with a recurrent carcinoma
**5.**	**Adjuvant chemotherapy colonic carcinoma (Process)**
*Numerator 5a*	Number of patients < 75 years old with a resected stage III (N1-2 M0) colonic carcinoma who received adjuvant chemotherapy
*Denominator 5a*	Number of patients < 75 years old with a resected stage III colonic carcinoma
*Numerator 5b*	Number of patients ≥ 75 years old with a resected stage III (N1-2 M0) colonic carcinoma who received adjuvant chemotherapy
*Denominator 5b*	Number of patients ≥ 75 years old with a resected stage III colonic carcinoma
*Inclusion*	All primary carcinomas, for which a part of the colon has been resected via open or laparoscopic surgery, and which have been
	classified as stage III in an postoperative pathology examination
*Exclusion*	1) patients who had a ‘resection’ via colonoscopy; 2) patients with a recurrent carcinoma
**6.**	**CRM rectum carcinoma (6a: Process, 6b: Outcome)**
*Numerator 6a*	Number of patients with a resected primary rectum carcinoma for which the CRM (circumferential resection margin) has been
	included in the pathology report and registered in the DSCA
*Denominator 6a*	Number of patients with a resected primary rectum carcinoma
*Numerator 6b*	Number of patients with rectum carcinoma with a CRM of 1 mm or less (tumor positive)
*Denominator 6b*	Number of patients with a resected primary rectum carcinoma
*Inclusion*	All patients who underwent a resection of rectum due to a primary rectum carcinoma in the reporting year, via open or
	laparoscopic surgery
*Exclusion*	TEM-resections and recurrent rectum carcinoma
**7.**	**Preoperative radiotherapy rectum carcinoma (Process)**
*Numerator*	Number of patients with T3 or T4 rectum carcinoma who received preoperative radiotherapy
*Denominator*	Number of patients with T3 or T4 rectum carcinoma
*Inclusion*	-
*Exclusion*	-
**8.**	**Volume (8a: Structure, 8b: Process)**
*Indicator 8a*	How many surgeons does the team include and how many of these surgeons carry out resections on primary colonic carcinoma
	patients?
*Indicator 8b*	Number of resections of primary colonic carcinomas
*Inclusion*	-
*Exclusion*	-

### Outcome measures

#### Quality indicators

The first outcome measure is the *computability* of quality indicators, and the corresponding results. Numerators and denominators of indicators are computable if all required items are available in a structured format.

As in [13] and [4], we analysed the accuracy of quality indicator results computed based on EMR data by measuring *sensitivity* and *specificity*. We also measure the *positive predictive value* (PPV) and the *negative predictive value* (NPV) as well as the *positive likelihood ratio* (PLR) and the *negative likelihood ratio* (NLR).

Whether the difference in proportions was significant has been tested with Bland’s and Butland’s method to compare proportions in overlapping samples [14]. A p-value < 0.05 was considered significant.

#### Data quality

We analysed the quality of the 14 data items required to compute the set of quality indicators (Operation date, Year of birth, Procedure, Operation urgency, Primary location/Diagnosis, cT score, pN stage, pM stage, Examined lymph nodes, Circumferential margin, Colonoscopy, Chemotherapy/Medication, Meeting date and Radiotherapy start date). The first quality dimension we analysed is *availability in a structured format*, as unstructured data cannot be used directly to automatically compute quality indicators. For data items that are available in a structured format, we focus on the quality dimensions *completeness* and *correctness*[15]. Completeness is measured as the percentage of items that should be recorded for each patient (such as the operation urgency, as all included patients have been operated) that are indeed available in the respective dataset. Items that do not necessarily apply to all patients, such as the start date of preoperative radiotherapy, are excluded, as a missing value might be due to the fact that the patient was indeed not treated with previous radiotherapy, but it might also be the case that the start date has not been recorded. Items explicitly recorded as ‘unknown’ are regarded as absent, diminishing completeness.

We measure *correctness* by checking whether data items recorded in the EMR are consistent with the corresponding items in the DSCA dataset with regard to the indicator definitions, i.e. whether they have the same effect on the indicator results. For example, a date for a multidisciplinary meeting is considered correct if both dates are before or both dates are after the operation.

Finally, encountered problems regarding data quality are categorised.

## Results

### Patient matching

**Figure 1:**
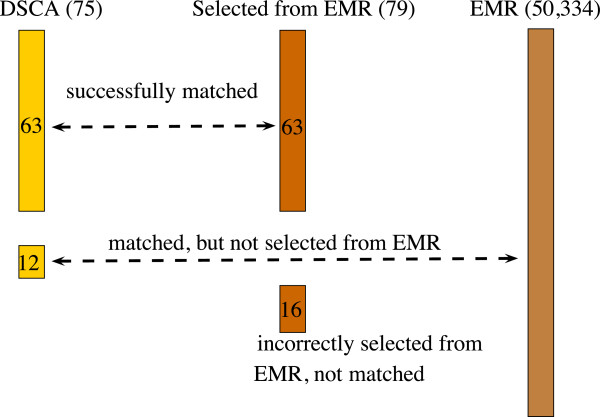
Matching of patients included in the DSCA dataset, selected from the EMR and included in the EMR.

As shown in Figure 1, 75 patients are included for the reporting year 2011 in the DSCA dataset, and 79 in the EMR. Following the matching strategy, it was possible to match all 75 DSCA patients with patients in the EMR. Sixty-three of these patients were also selected by the query to compute the indicators based on the EMR dataset, while 12 patients were not selected. Manual inspection showed that 4 of these 12 patients had no relevant diagnosis recorded in the EMR. A fifth patient was recorded with a colonic carcinoma and a resection of rectum, but the query against the data warehouse selected patients with a colonic carcinoma and colectomy or a rectum carcinoma and resection of rectum. For the remaining 7 patients, the diagnosis date was after the (elective) operation date, so that a relationship between diagnosis and operation could not be assumed.

Sixteen patients from our EMR dataset could not be matched to the DSCA dataset because they were selected incorrectly due to incorrect (e.g. tumours that were classified as non-malignant based on the pathology examination) or imprecise (e.g. recurrent carcinomas) diagnosis codes or despite missing relations between the diagnosis and the procedure in the EMR dataset.

### Computation of quality indicators

Table 2 shows the indicator results computed based on the DSCA dataset, as well as fully computable indicators and denominators based on the EMR data. The chemotherapy indicators 5a and 5b as well as the radiotherapy indicator 7 could not be computed, as the required carcinoma’s stage was not available in a structured format.

**Table 2:** Indicator results based on both datasets

**Indicator**	**DSCA**	**EMR**	**Sensitivity**	**Specificity**	**PPV**	**NPV**	**PLR**	**NLR**
**1 DSCA**	(75/-)	(-/79)	-	-	-	-	-	-
2 lymph nodes	85% (39/46)	(-/36)	-	-	-	-	-	-
3 meeting	100% (29/29)	70% (23/33)	79% (23/29)	- (0/0)	100% (23/23)	0% (0/10)	-	-
4 imaging	88% (36/41)	58% (31/53)	58% (21/36)	60% (3/5)	67% (21/31)	14% (3/44)	1,45	0,7
5a chemotherapy	80% (8/10)	-	-	-	-	-	-	-
5b chemotherapy	17% (1/6)	-	-	-	-	-	-	-
**6a CRM**	62% (18/29)	(-/33)	-	-	-	-	-	-
6b CRM	14% (4/29)	(-/33)	-	-	-	-	-	-
7 radiotherapy	92% (22/24)	-	-	-	-	-	-	-
8b volume	46	37	-	-	-	-	-	-

Percentages are denoted as % (numerator/denominator). The bold indicators are those for which only the denominator has been included.

#### Comparison of selected patients

Table 3 shows the comparison of selected patients for all fully computable indicator elements.

**Table 3:** Patients selected based on the two datasets

**Indicator**	**Element**	**DSCA**	**EMR**			
			**EMR**	**TP (DSCA and EMR)**	**FP (DSCA only)**	**FN (EMR only)**
1 DSCA	Num/denom	75	79	63	12	16
2 nodes	Denominator	46	36	28	18	8
3 meeting	Numerator	29	23	23	6	0
3 meeting	Denominator	29	33	25	4	8
4 imaging	Numerator	36	31	21	15	10
4 imaging	Denominator	41	53	31	10	22
6a and 6b CRM	Denominator	29	33	25	4	8
8b volume	-	46	37	28	18	9

TP stands for True Positives, FP for False Positives and FN for False Negatives.

### Outcome measures

#### Quality indicators

All 10 indicators were fully computable based on the DSCA dataset. Eight of these indicators should in principle be fully computable based on EMR data, but in practice this was the case for only three indicators. For the two indicators (multidisciplinary meeting and imaging) that are percentages, the difference in proportions computed based on the two datasets was significant.

For 4 indicators, only the denominators were fully computable, because the data items defining the quality of care measured in the numerator, such as the number of examined lymph nodes, were not available in a structured format.

#### Data quality

The results of the data quality analysis are given in Table 4. Fourteen data items are required to compute the set of quality indicators. All of these items are available in the DSCA register, and 8 in the EMR, with the remaining 6 only being available in free text. The pathology reports contained in the EMR comprise required data such as the number of examined lymph nodes, the circumferential margin and the pathological stage of the carcinoma only in free text. The clinical stage of the carcinoma is equally unavailable, although it might be present in free text sources that we did not have at our disposal, such as conclusions of physical or radiologic examinations or endoscopies, or contained in referral letters. It is contained in a structured format in the Dutch Cancer Registry, but the goal of our study was to focus on the data in our EMR.

**Table 4:** Data quality

**Item**	**Completeness DSCA**	**Completeness EMR**	**Correctness**
Operation date	100% (75)	100% (75)	100% (75)
Year of birth	100% (75)	100% (75)	100% (75)
Procedure	100% (75)	100% (75)	97% (73/75)
Operation urgency	100% (75)	100% (75)	95% (71/75)
Primary location/Diagnosis	100% (75)	100% (75)	91% (68/75)
cT score	39% (29)	0% (unavailable)	-
pN stage	100% (75)	0% (unavailable)	-
pM stage	100% (75)	0% (unavailable)	-
Examined lymph nodes	99% (74)	0% (unavailable)	-
Circumferential margin	24% (18)	0% (unavailable)	-
[Colonoscopy]	[100% (75)]	[80% (60)]	83% (50/60)
[Chemotherapy/Medication]	[99% (74)]	[97% (73)]	21% (15/73)
[Meeting date]	[85% (64)]	[79% (59)]	98% (57/58)
[Radiotherapy start date]	[33% (25)]	[24% (18)]	100% (18/18)
Average of available items	86%	50%	87%

Elements enclosed by square brackets are not supposed to be available for each patient.

For data items that should be recorded for each patient, the average completeness is 86% for the register’s dataset and 50% for the EMR. The average correctness of data items in the EMR is 87%.

### Catalogue of encountered problems

In our case study, quality indicators could not be computed reliably based on the EMR data due to the general problems as enlisted in Table 5.

**Table 5:** Catalogue of encountered problems

**Problem**	**Explanation**
Data not available in	Data items required to compute many of the indicators, such as those contained in the pathology reports, were only
structured format	available in non-structured free text, and therefore not directly (re)usable. Also structured data to exclude patients based on
	the exclusion criteria *recurrent carcinoma* and *TEM-resection* as well as *‘resection’ via colonoscopy* was not available in our EMR
	nor in the DSCA dataset. Non-recorded exclusion criteria can lead to lower indicator results, wrongly underestimating the
	quality of care for indicators whose percentages are to be maximised [16,17].
Incorrect data items	The double data entry in our case study helped us to discover incorrect data items. Furthermore, we identified imprecise
	and/or incorrect diagnosis codes in our EMR.
Incomplete view of	Hospitals throughout the country refer patients to our hospital, which specialises in gastro-intestinal oncology. Some of
patient history	these patients are only treated for a short time, and then referred back. Likewise, our hospital maintains an alliance with a
	nearby hospital. Referral letters are typically posted as physical letters, making a complete, consistent view on a patient’s
	history difficult to obtain. For example, it is hard to retrace whether preoperative imaging of the colon has taken place in
	another hospital.
Lack of relations	Our EMR does not store any relations between diagnoses and procedures, making it impossible to select the diagnosis that
between data items	was the underlying reason for a procedure. For example, the lymph node indicator should only select lymph node
	examinations that have been carried out in the context of a primary colonic carcinoma, and not, for example, a previous
	mamma carcinoma. As a partial solution, we imposed the constraint that the diagnosis should have been established before
	the related operation was carried out, which resulted in some missed patients.
Lack of detail	None of the diagnoses in the EMR was detailed enough to meet the information required by the indicators, which include
	patients with *primary* colonic and rectum carcinomas. The only relevant diagnoses in the EMR were malignant neoplasm
	of colon, rectum and rectosigmoid junction. Therefore, the concepts employed in the queries to compute the indicators
	had to be generalised. Furthermore, only the type of endoscopies is registered, such as colonoscopy, but not whether the
	complete colon is affected.
Lack of standardisation	For example, the urgency of an operation is defined in the EMR according to 8 categories, but the DSCA dataset only
	differentiates urgencies according to 4 categories. It was not clear how these categories should be mapped, as their
	meaning was not unambiguously described (for example, one of the categories was called “extra”).

## Discussion

Our results show that EMR-based indicator results significantly underestimate the quality of care compared to the same indicators computed based on manually abstracted data for a national quality register. Reasons were unavailable, incomplete and incorrect data items as well as missing relationships between diagnoses and procedures in the EMR. In particular, detailed data that reflects whether a patient’s treatment met the ideal standard of care was often incomplete in the EMR.

### Comparison with other studies

The use of EMRs has increased rapidly in the recent years, making trustworthy reuse of data [18] an important challenge and research question. Worldwide, EMR-based quality measures [19] are increasingly employed, and new standards [20] such as *eMeasures* to automatically derive quality measures from EMRs are introduced.

Many researchers have compared results computed based on different data sources. Both Kerr et al. [21] and Parsons et al. [22] found that EMR-derived measures can underestimate performance in comparison to manual abstraction. Kern et al. [4] found that a “wide measure-by-measure variation in accuracy threatens the validity of electronic reporting”. Likewise, results of quality indicators computed based on administrative data have been compared to results computed based on manually abstracted EMR data. MacLean et al. [23] found that the EMR allows for a greater spectrum of measurable quality indicators, while summary estimates computed based on both data sources did not differ substantially. Tang et al. [24] found a significantly higher percentage of patients that have been identified to be relevant by manual selection.

Ancker et al. observed that “secondary use of data […] requires a generally higher degree of data integrity than required for the original primary use” [25]. It has been suggested that reliable and valid quality indicator results are only achievable based on accessible and high-quality data [26-33]. Likewise, it has been shown that data quality issues are common in data warehouses and electronic patient records [34-36].

### Limitations of this study

Our case study included one hospital and one year of data with a relatively small sample size, and it is questionable to what extent the situation in our hospital is generalisable to other hospitals. However, the sample size was sufficient to show that data quality can significantly influence computed quality indicator results, which should be independent from the respective location.

### Recommendations/future work

Based on the encountered problems, we compiled a set of recommendations to improve the quality and (re)usability of EMR data.

#### Availability of structured data

Data to determine the quality of care is particularly valuable, and hospital information systems should be set up in such a way that this data is available, accessible and usable for quality measurement and further use-cases. To obtain structured data, synoptic reports, i.e. predefined computer-based forms to record relevant procedures and findings in a structured, standardised format, have been shown to be advantageous [37-39]. A standard way to encode medical free text is the use of Natural Language Processing tools. However, as most tools are developed for English, further research is required to handle Dutch [40].

#### Correctness of data items

Multiple data entry is unnecessary, error-prone, tedious and time-consuming. Data should be recorded only once, in an adequate quality. The quality might be risen by making those entering data aware of its possible reuses. Also, local quality improvement strategies from the literature [7,41] could be applied. To submit data to the DSCA under such improved circumstances, required items could be preselected automatically from the EMR, checked by the one responsible and be submitted to quality registers or other authorised parties. If the data needs to be edited, changes should be applied locally before the data is shared with external parties.

#### Longitudinal view of patient history

As patient referrals are common and hospital alliances are likewise to proliferate in the future, it must become common practice to exchange data securely and automatically. Patients are likely to become active managers of their health, increasingly enabled to share their data with their caregivers.

#### Relations between diagnoses and procedures

To reuse clinical data, the relations between diagnoses and procedures must be traceable. To be able to automatically select only examinations that have been carried out in the context of a certain diagnosis, such relations should be recorded.

#### Level of detail

Patient data should be recorded as detailed as necessary for quality indicator computation and further foreseeable use-cases, such as the recruitment of patients for clinical trials, decision support, the early detection of epidemics or general clinical research. This might seem time-consuming, but will likely reduce the workload in the long term, as each data item has to be recorded only once. To further reduce the workload, the process should be supported by advanced data entry methods and interfaces.

#### Standardisation

Only data that is represented meaningfully - ideally in standard codes from comprehensive controlled clinical terminologies - can be reused automatically. Terminologies such as SNOMED CT can support the “Collect once - use many times” paradigm [42], which stands for the idea that data is captured only once and can be reused thereafter for a variety of purposes. Controlled terminologies can allow for meaning-based retrieval, for example by aggregation along hierarchical structures, or based on relationships between codes. An advantage of standard terminologies is that they are integrated in the National Library of Medicine’s Unified Medical Language System Metathesaurus, which contains mappings between terms across multiple terminologies.

## Conclusions

This study showed that data quality can significantly influence indicator results, and that our routinely recorded EMR data was not suitable to reliably compute quality indicators. To support primary and secondary uses of data, EMRs should be designed so that a core dataset consisting of relevant items is entered directly and timely in a structured, sufficiently detailed and standardised format. Furthermore, awareness about the (re)use of data could be risen to ensure the quality of required data, and local data quality improvement strategies could be applied. Data could then be aggregated for different uses, according to various definitions. This strategy likely leads to an increased volume of high-quality data, which can ultimately serve as a basis for physicians not only to monitor but also to deliver the best possible quality of care.

## Competing interests

The authors declare that they have no competing interests.

## Authors’ contributions

KD, RC, AtT and NdK conceived and designed the study. KD conducted the analysis and wrote the first draft of the paper. PT entered and interpreted the data. All authors reviewed the manuscript and approved the final version. KD is guarantor for the study.

## Pre-publication history

The pre-publication history for this paper can be accessed here:

http://www.biomedcentral.com/1472-6947/14/32/prepub
